# Industrially scalable surface treatments to enhance the current density output from graphite bioanodes fueled by real domestic wastewater

**DOI:** 10.1016/j.isci.2021.102162

**Published:** 2021-02-07

**Authors:** Emma Roubaud, Rémy Lacroix, Serge Da Silva, Jérôme Esvan, Luc Etcheverry, Alain Bergel, Régine Basséguy, Benjamin Erable

**Affiliations:** 1 Laboratoire de Génie Chimique, Université de Toulouse, CNRS, INPT, UPS, Toulouse, France; 2 6T-MIC Ingénieries, 9 rue du développement – ZI de Vic, 31320 Castanet-Tolosan, France; 3 Cirimat, Université de Toulouse, CNRS-INP-UPS, 4 allée Emile MONSO, BP 44362, 31030 Toulouse, France

**Keywords:** Electrochemistry, Microbiofilms, Surface Science

## Abstract

Acid and electrochemical surface treatments of graphite electrode, used individually or in combination, significantly improved the microbial anode current production, by +17% to +56%, in well-regulated and duplicated electroanalytical experimental systems.

Of all the consequences induced by surface treatments, the modifications of the surface nano-topography preferentially justify an improvement in the fixation of bacteria, and an increase of the specific surface area and the electrochemically accessible surface of graphite electrodes, which are at the origin of the higher performances of the bioanodes supplied with domestic wastewater. The evolution of the chemical composition and the appearance of C-O, C=O, and O=C-O groups on the graphite surface created by combining acid and electrochemical treatments was prejudicial to the formation of efficient domestic-wastewater-oxidizing bioanodes. The comparative discussion, focused on the positioning of the performances, shows the industrial interest of applying the surface treatment method to the world of bioelectrochemical systems.

## Highlights

• Electromodified graphite promoted the ESEA and the fixation of bacteria
• Surface electromodification increased mostly the surface nanotopography and capacitance
• Bioanodes fed only with domestic wastewater continuously supplied 2.8 A/m^2^
• The predominance of bacteria did not differ despite the surface modification

## Graphical abstract

**Figure:**
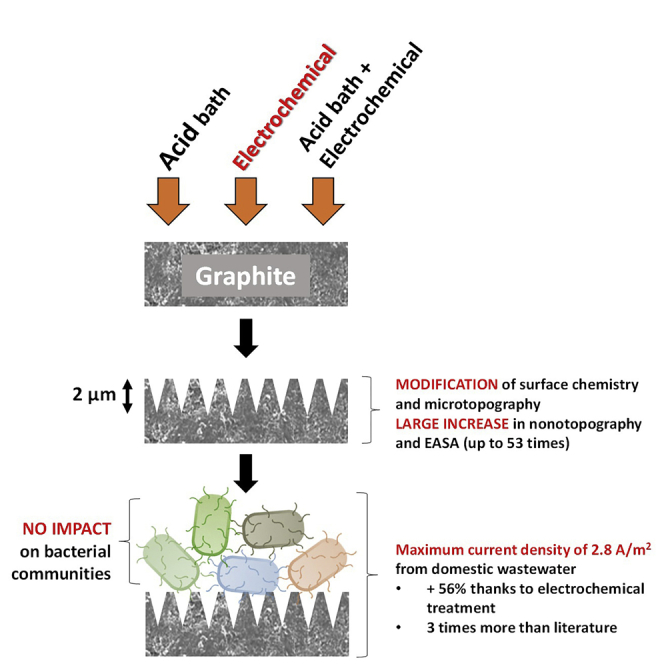

## Introduction

Bio-electrochemical technologies such as microbial electrolysis cells (MECs) and microbial fuel cells (MFCs) are promising processes for wastewater energy recovery (Zhu et al., 2011). However, the deployment of these technologies is (generally) limited by the low oxidation kinetics of the bioanode, especially when low-COD (chemical oxygen demand) effluents such as domestic wastewater (dWW) are treated (Liu et al., 2014).

Among possible ways of boosting the anodic reaction process, one option to increase the oxidation kinetics and current densities generated by bioanodes is the surface treatment of the bioanode materials (Li and Cheng, 2019; Mohamed et al., 2018). Electrode surface treatments modify several aspects of the bioanodic reaction (Li and Cheng, 2019; Mohamed et al., 2018), by improving extracellular electron transfer (EET), increasing the specific surface area of the anode, or promoting the adhesion of bacteria that initiate the formation of electroactive biofilms, for example.

Surface modification of electrode materials can be achieved by chemical, electrochemical, mechanical, thermal, pressure, or vacuum treatment or a combination of these methods. A distinction is made between methods in which exogenous materials can be added (coating, grafting, painting, etc.) and those that do not involve any additional material (passivation, polishing, machining, etc.). Using modified carbon electrodes is a new approach for the development of bioelectrochemical systems. Table 1 summarizes the major surface treatments that, applied to carbon-based materials, have significantly improved the current densities of bioanodes. All the methods cited are included in this literature review, particular attention being paid to the features necessary to make them widely available at an industrial scale, i.e., simplicity of implementation, low cost, rapidity of execution, and the possibility of benefiting from existing surface treatment industries. *In fine*, significant gains, ranging from 20% to 256%, have been reported depending on the anode pre-treatment methods used (Fiset and Puig, 2015).

**Table 1:** Effect of surface treatments applied to carbon-based anode materials and consequences for current density improvement in microbial bioanodes

Type of treatment	Anodic material	Effect on the surface	Untreated electrode current density	Treated electrode current density	Current density improvement	Reference
Acid bath (nitric acid)	Activated carbon fibers	Increases the number of N functional groups on the surface, improves bacterial adhesion	6 A/m^2^	9.5 A/m^2^	58%	(Zhu et al., 2011)
Acid bath (formic acid)	Carbon cloth	Decreases the number of O and N functional groups on the surface, increases EET rate	3.6 A/m^2^	4.8 A/m^2^	33%	(Liu et al., 2014)
Doping (Ag nanoparticles)	Graphite plate	Increases the specific surface area and improves EET rate	1,100 A/m^3^	1,400 A/m^3^	27%	(Sadri et al., 2017)
Doping (Ca-S particles)	Activated carbon granules	Improves bacterial adhesion	23.1 A/m^3^	40.1 A/m^3^	74%	(Yasri and Nakhla, 2017)
Electrochemical (applied voltage)	Graphite plate	Increases porosity and electrochemical capacitance	1.6 A/m^2^	2.6 A/m^2^	63%	(Tang et al., 2015)
Electrochemical (applied potential)	Carbon felt	Creates micro-cavities on the surface, improves bacterial adhesion	450 mA/m^2^	1,600 mA/m^2^	256%	(Cercado-Quezada et al., 2011)
Electrochemical (applied current) + acid bath (nitric + sulfuric acids)	Carbon cloth	Increases the number of functional groups on the surface, decreases anode resistance to EET, and improves bacterial adhesion	153 μA/cm^2^	183 μA/cm^2^	20%	(Li et al., 2014)
Mechanical (roughened surface)	Graphite plate	Increases the specific surface area	33 A/m3	43 A/m3	30%	(Ebrahimi et al., 2017)
Hydrothermal	Graphite plate	Increases the specific surface area and the wettability, improves bacterial adhesion and EET rate	520 mA/m^2^	990 mA/m^2^	90%	(Liu et al., 2017)

Electrochemical treatment and acid bath treatment are among the simplest industrial processes for surface treatment, and their combination is technologically easy to achieve. In addition, through experiments on MFCs, Li et al. (2014) have already validated the advantages of coupling electrochemical and acid pre-treatments of carbon cloth anodes. The start-up of their MFCs integrating treated carbon cloth anodes was accelerated due to a stronger and faster adhesion of the anodic biomass. Above all, the long-term power and current generation was significantly increased (by 20%) compared with those of MFCs integrating bioanodes based on untreated commercial carbon cloth bioanodes. This pioneering work highlights the major effect that modifying the surface chemistry of the material has on the electrical resistance of the material, on the modification of the electrochemically accessible surface area (EASA), and, finally, on the bacterial fixation. However, additional questions persist concerning the possible consequences of this type of surface modification on the interactions of the material with electrolytes carrying soluble molecules, colloids (macromolecules), particles, and various other microbial cells. It is now well known in the broad field of biofilms (Carniello et al., 2018; Wu et al., 2018), and more specifically in that of electroactive biofilms (Champigneux et al., 2018b), that the topography and chemistry of solid surfaces play essential roles in the early stages of biofilm formation (detection, adsorption, adhesion, and anchoring). It is therefore logical to infer that the microbial biofilms forming on carbon electrodes whose surfaces have been modified at several scales in terms of morphology and chemical composition may be different and have singular electrocatalytic properties.

In the present study, we therefore focused on understanding the individual and combined effects of electrochemical anodization (Cercado-Quezada et al., 2011) and acid treatment (Li et al., 2014) on the modification of flat graphite electrode surfaces and the consequences they generate regarding microorganism-material interactions and the electrocatalytic properties of biofilm-material interfaces. The effects of the treatment protocols on the graphite surface were first evaluated from the standpoints of changes in topography (roughness [Pocaznoi et al., 2012a], specific surface [Roberts and Slade, 2010]), evolution of the elemental and chemical composition (X-ray photoelectron spectroscopy [XPS]), and improvement of the electrochemical reactivity (material capacitance, electrochemically active surface). Then the treated electrodes were compared, under electroanalytical conditions (electrode potential and fuel concentration maintained at constant values [Rimboud et al., 2014; Roubaud et al., 2019]), for their ability to act as dWW-fueled bioanodes. The current densities produced were compared with those of the control bioanodes (i.e., untreated graphite electrodes). Finally, the biodiversity of the bacterial communities established on the surface of both treated and untreated materials was determined by a molecular inventory of bacterial species (Lu et al., 2012; Mateo et al., 2018).

## Results

### Surface topography modifications resulting from acid, electrochemical, and combined treatments

**Figure 1:**
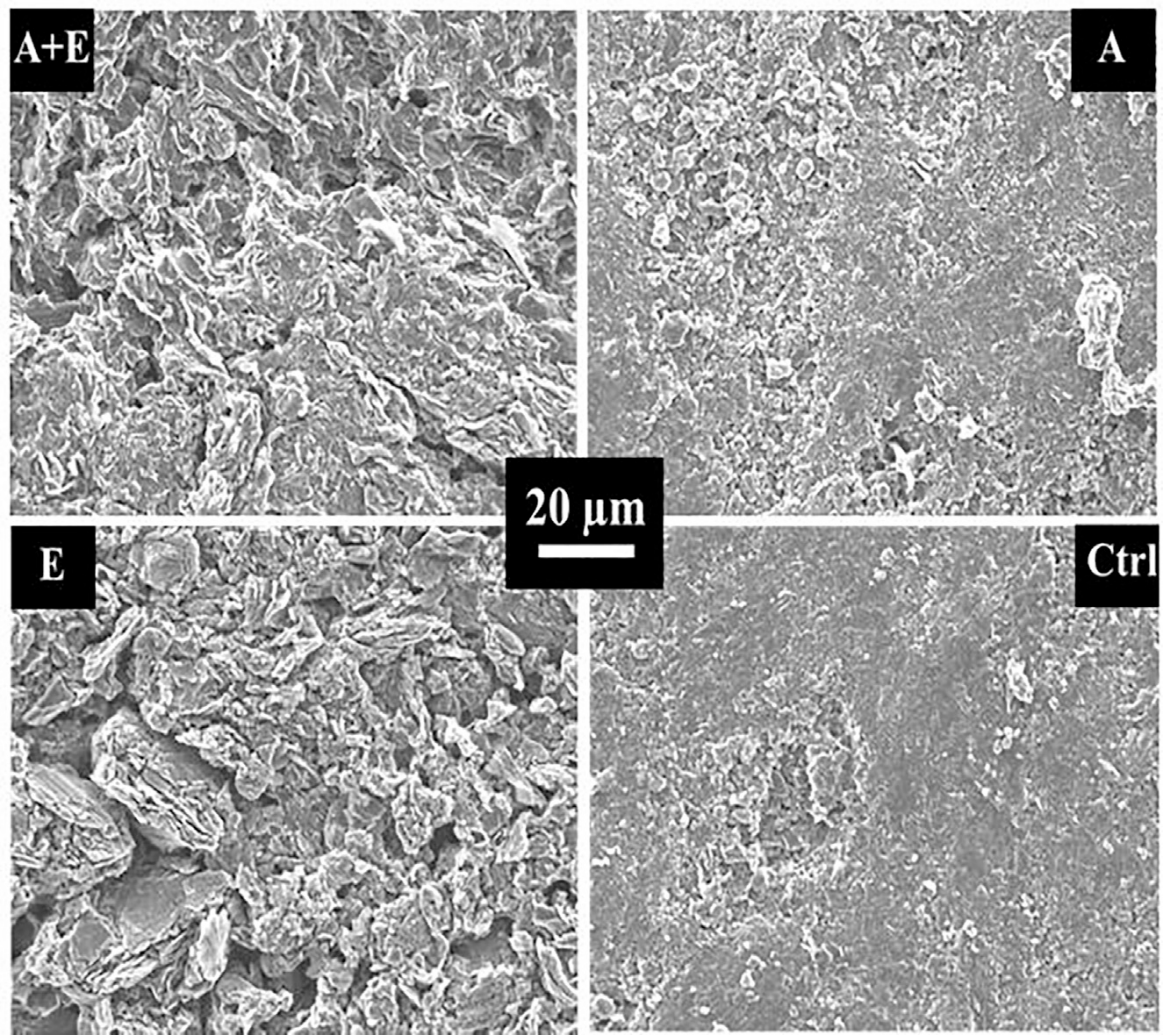
SEM images of treated graphite surfaces (A + E, A, E) and non-treated commercial graphite surface (Ctrl) A, acid; E, electrochemical; Ctrl, control.

After the treatments had been performed on plain graphite electrodes following the acid bath treatment (A), electrochemical treatment (E), and A + E protocols, electrode surfaces were first imaged by scanning electron microscopy (SEM) to detect the main topographic microscale changes, such as roughness, sharpness, deposits, and damage of the surface. SEM images clearly showed substantial modifications of the surface topography on the graphite that underwent E and A + E treatments compared with the control electrode (without graphite treatment) (Figure 1). In more detail, as a result of the E and A + E treatments, the surface showed very sharply cracked graphite layers, reminiscent of the graphene layers obtained by Tang et al. (2015), also on a graphite plate, through electrochemical exfoliation with a direct current of +10 V applied to the plate for 5, 15, or 40 min (Tang et al., 2015). The presence of graphene layers extended the porosity and electrochemical double layer capacitance of the graphite, leading to higher current densities of the microbial bioanodes formed with this engineered material. The application of the acid treatment alone seemed to modify the topography of the graphite only slightly; the surface simply became a little more irregular.

Two roughness parameters were then quantified by optical microscopy: S_a_, which is the arithmetic mean roughness, and S_ku_, which measures the sharpness of the surface. The sharpness increases when the S_ku_ decreases (Champigneux et al., 2018a). S_a_ and S_ku_ measurements for the raw graphite surface or surfaces modified by acid, electrochemical, or combined treatments are given in Table 2. S_a_ increased by 18% for the electrode surfaces modified by treatments E and A + E compared with the surface of the control electrode. Surprisingly, S_a_ decreased by 8% for the electrode with treatment A, whereas the interpretation of the SEM image rather suggested that the surface was a little rougher. S_ku_ values decreased by 70% after treatments A + E and E and by 65% after treatment A. All treatments significantly increased the sharpness of the surface with respect to the control electrode. These changes in sharpness are also clearly visible in the SEM images for the A + E and E electrodes, but are still not as clearly interpretable for the A electrode.

**Table 2:** Surface roughness parameters for treated (A, E, and A + E) and non-treated (control) graphite electrodes

Graphite electrode		A + E	A	E	Control
**Surface roughness parameters**	**S**_**a**_**(μm)**	2.9 ± 0.1	2.3 ± 0.1	2.9 ± 0.1	2.5 ± 0.3
	**S**_**ku**_	6 ± 1	7 ± 1	6 ± 1	20 ± 3

A, acid; E, electrochemical. Parameters are represented as the mean and the standard deviation of 10 test data on the graphite surface.

### Influence of the treatment on graphite electrode specific surface areas

The determination of the specific surface areas of graphite treated by the classical Brunauer-Emmett-Teller (BET) gas adsorption method and untreated graphite showed a qualitative increase in the specific surface area of graphite after the A + E and E treatments (Table 3). The specific surface area measured by the BET method was 2.7 m^2^/g and 2.1 m^2^/g for electrodes A + E and E, respectively, whereas the specific surface areas of the control and A-treated electrodes were below the measurement limit of 1 m^2^/g quantifiable by the Belsorp-Max system equipment.

**Table 3:** Electrochemically accessible surface area and specific surface area determined for treated (A, E, and A + E) and non-treated (control) graphite electrodes

Graphite electrode	A + E	A	E	Control
Specific surface area (m^2^/g)	2.7	<1	2.1	<1
EASA (cm^2^)	214	34	171	4

A, acid, E, electrochemical; EASA, electrochemically accessible surface area.

<1: less than quantitative limit of analytical method.

**Figure 2:**
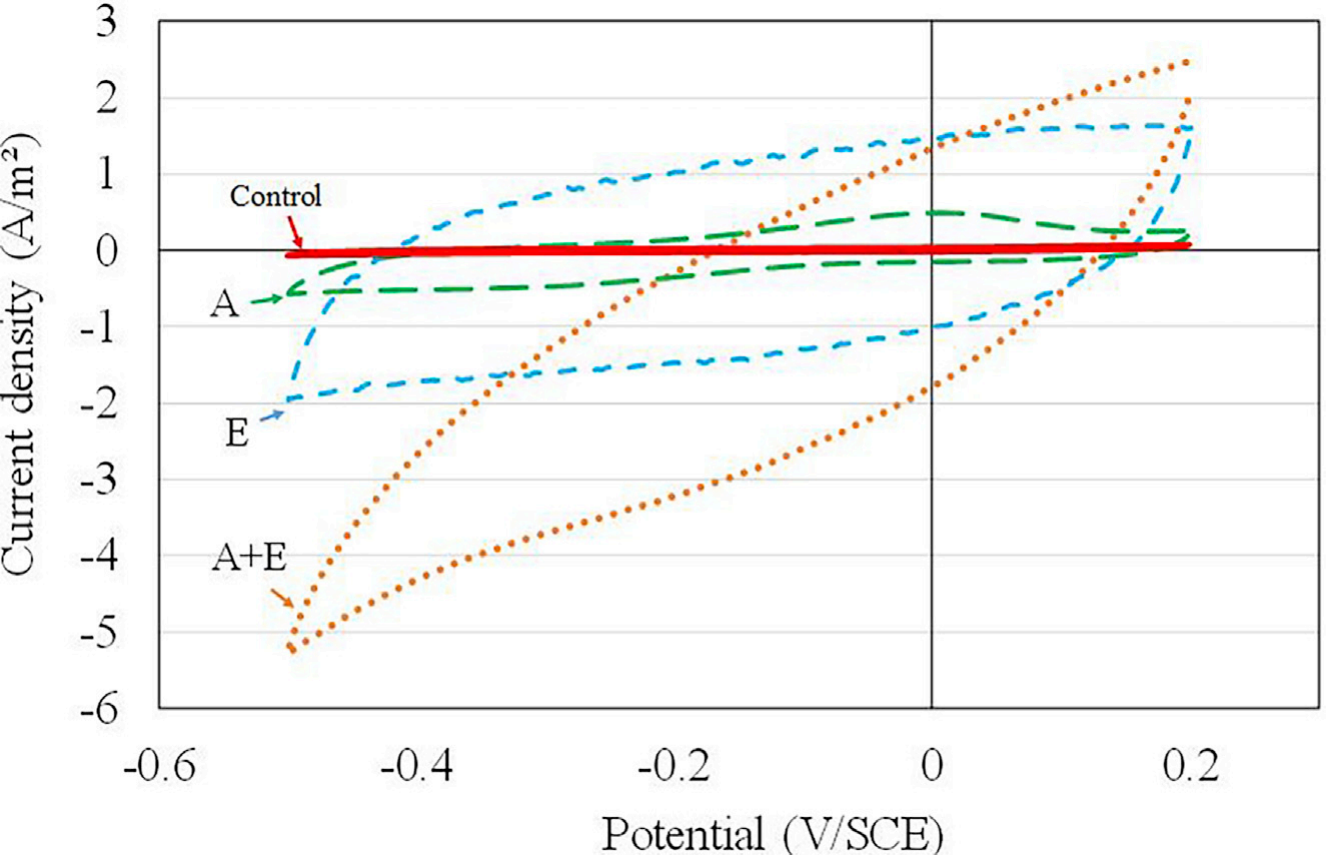
Cyclic voltammetries performed in dWW on treated graphite surfaces (A + E, A, E) and non-treated commercial graphite surface (Control) before microbial colonization Scan rate: 1 mV/s. A, acid; E, electrochemical.

To understand in greater detail and to evaluate the consequences of the treatment on the surface electrochemical reactivity in operating conditions, cyclic voltammetry (CV) was performed with dWW as the electrolyte solution on previously treated and untreated 4-cm^2^ non-colonized graphite electrodes (Figure 2). Compared with the untreated control electrode (Control), all electrodes that had been subjected to acid and/or electrochemical treatment (A, E, A + E) presented voltammograms with a rectangular shape characteristic of capacitive behavior. In addition, the A + E electrode also exhibited high resistive properties, indicated by the inclination of the curve I/E with respect to the abscissa axis. This graphical representation actually appeared to follow a classical Ohm's law relationship, the average current density being almost directly proportional to the electrode potential.

The material capacitance was calculated using the following equation:

$$\;J_{C}=\frac{J_{ox}-J_{red}}{2}=\;v\;.\;C$$

where J_C_ is the capacitive current that corresponds to the half-width of the pseudo-rectangular part of the voltammogram, J_ox_ and J_red_ are the currents measured at a given potential during the CV plotting in the oxidation and reduction directions, respectively; *v* is the scan rate (V/s); and C is the capacitance of the electrode (F).

The capacitance values, calculated on almost the whole potential range explored, were 632 mF, 504 mF, 100 mF, and 12 mF for the A + E, A, E, and control electrodes, respectively, clearly showing that the treatment increased the surface capacity to collect and stock charges in the electrochemical double layer.

If it is assumed that the increase of the capacitance was due to an increase of the specific surface area and of the surface porosity of the graphite; the EASA, which is the electrochemically accessible electrode surface area, can be calculated based on the capacitive current using the following equation (Li et al., 2014):

$$EASA=\frac{C}{C_{sp}}=\;\frac{J_{C}}{v}.\frac{1}{C_{sp}}$$

where C_sp_ is the specific capacitance of the control graphite electrode (F/m^2^). Thus the calculation of the EASA was predicated on the prior determination of the electrochemical double layer specific capacitance (*Csp*) of the control electrode. Assuming a control electrode area of 4 cm^2^, the *Csp* was evaluated at 29.5 F/m^2^. The EASA of treated electrodes A + E, E, and A are reported in Table 3. With respect to the control electrode, the EASA was multiplied by 54 after the A + E treatment, by 43 after the E treatment, and by only 8 after the A treatment. In this last case (A treatment), the increase of the specific surface area was not enough to be accurately, quantitatively measured by the BET method. However, the results obtained for the experimental quantification of EASA and BET measurements of surface area appear to be relatively consistent with each other and support our argument that the increase in EASA is the consequence of an increase in surface area after treatments.

### Surface chemistry modifications

XPS analysis (Table 4) showed only slight surface chemistry modifications after A and E treatment of graphite electrodes. The percentage of O ranged from 13.0% for the control to 15.0% and 16.0% after A and E treatments, respectively. More significant chemical modifications appeared with the A + E treatment. The O percentage rose from 13.0% to 25.7%, and the N percentage from 0.5% to 2.6%. The oxidation phenomenon occurring with the A + E treatment can be observed by the increase in the percentage of C-O, C=O, and O=C-O groups. However, the same surface chemistry modifications were not observed after the E treatment. The modifications may be linked to the pH of the electrolyte in which the cyclic voltammetry (CV) was performed: acid medium (pH was −0.6) due to the presence of H_2_SO_4_/HNO_3_ for A + E treatment, instead of mild pH (phosphate electrolyte) for E treatment. In acidic conditions, C-O, C=O, and O=C-O groups are more easily formed on the graphite surface as the O_2_ production potential is higher than in pH-neutral conditions. In pH-neutral conditions, a higher oxygen production could limit the production of those functional groups.

**Table 4:** XPS analysis of elemental composition of treated graphite surfaces (A + E, A, and E) and non-treated commercial graphite surface (control)

Electrode	A + E	A	E	Control
**C**	68.5	80.9	81.3	83.6
**O**	25.7	15.0	16.0	13.0
**N**	2.6	0.5	0.6	0.5
**S**	3.0	3.1	1.7	2.7
**P**	0.2	0.5	0.4	0.2
**C-C; C-H; C=C**	47.3	71.8	71.3	74.6
**C-O; C-N**	13.4	6.0	5.7	7.1
**C=O**	2.5	1.4	0.8	0.6
**O=C-O**	5.3	1.7	3.5	1.3

First five lines: atomic % of each element, last four lines: distribution of the carbon content. A, acid; E, electrochemical.

### Performance of microbial bioanodes fueled by domestic wastewater

Microbial bioanodes were produced at a constant potential of −0.1 V/SCE (i.e., electroanalytical system) from treated (A, E, and A + E) and non-treated (control) graphite electrodes, using the strict comparison method detailed and validated in Roubaud et al. (2019) (Roubaud et al., 2019). Two successive experimental runs were performed with two different batches of dWW and activated sludge (AS) to duplicate the experimental results. The same batch of dWW and AS was used in the four reactors of a given run, but two different batches where used for run 1 and run 2. Monitoring of the exchange current obtained with the treated and control graphite electrodes over 25 days gave the curves shown in Figure S1.

The steady-state current densities (simply noted “current densities” in the rest of the text) averaged over the current densities recorded in the chronoamperometry (CA) between days 10 and 25 for each reactor are shown in Table 5. All surface treatments (A, E, A + E) of the graphite electrodes resulted in the generation of microbial bioanodes delivering significantly higher current densities than that produced by the control electrode. Compared with the control bioanode, the average current densities produced by the A + E and A electrodes were 17% higher and those with the E electrodes were 56% higher. The current densities obtained with the first series of experiments were 20%–35% lower than those obtained with the second series for all experiments, no particular link being observed with the A + E, E, or A electrodes, or the control electrodes. This difference in performance between the two series could simply have been caused by the origin of the AS used as inoculum between series 1 and 2 or the temperature, which was not thermostatically controlled during the experiments.

**Table 5:** Steady state current densities produced by dWW-fueled microbial bioanodes produced from treated (A, E, and A + E) and non-treated (control) graphite electrodes

Electrode	A + E	A	E	Control
Run 1 (A/m^2^)	1.7 ± 0.5	1.7 ± 0.3	2.2 ± 0.6	1.6 ± 0.5
Run 2 (A/m^2^)	2.4 ± 0.4	2.5 ± 0.4	3.4 ± 0.6	2.0 ± 0.2
**Average over both runs (A/m**^**2**^**)**	2.1	2.1	2.8	1.8
**Current density improvement**	+17%	+17%	+56%	NA

Steady state current densities were calculated by averaging the current density values recorded from the first day of current generation stability (10^th^ day) to the end of the experiment (25^th^ day). A, acid; E, electrochemical.

**Figure 3:**
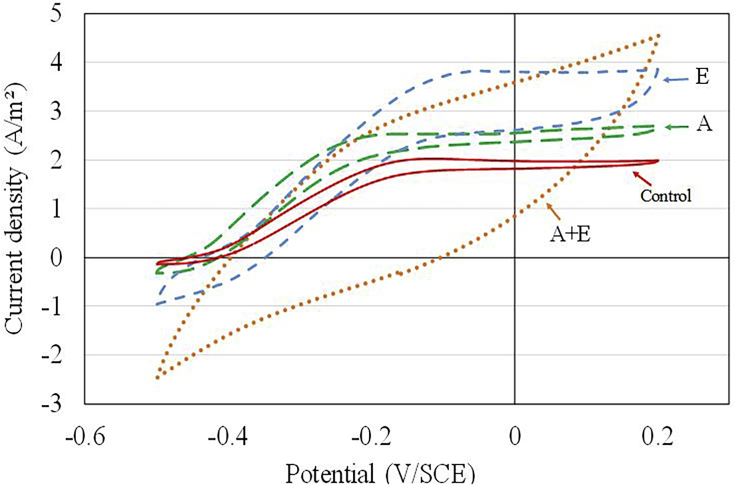
Cyclic voltammetry performed in dWW on treated graphite surfaces (A + E, A, E) and non-treated commercial graphite surface (Control) after microbial colonization (end of the 25-day experiment) Scan rate: 1 mV/s. A, acid; E, electrochemical.

After 25 days of constant electrode polarization at −0.1 V/SCE, turn-over CVs were performed in dWW containing a concentration of 360 mg COD/L (Figure 3). This concentration has already been experimentally validated to allow *Jmax* to be reached without dependence on the concentration of organic matter (Roubaud et al., 2019).

The A, E, and control electrode CV graphs displayed a shape characteristic of bioanodes oxidizing volatile fatty acids that mainly represent soluble COD (acetate, formate, butyrate, and propionate [Barker et al., 1999]), at neutral pH (Cercado-Quezada et al., 2013; Pocaznoi et al., 2012b). The half-wave potential was around −0.30 V/SCE for the A and control bioanodes and −0.25 V/SCE for the E bioanode. The current plateau started from approximately −0.2 V/SCE for A and control microbial bioanodes, and from −0.1 V/SCE for the E microbial bioanode. The E-treated microbial bioanode also displayed highly capacitive behavior (C = 248 mF), whereas the capacitive current of the A bioanode was identical to that of the control bioanode (46 mF). This was not the case before microbial colonization of the anodes. On the contrary, the CV graph obtained with the A + E microbial bioanode showed a strongly capacitive (C = 627 mF) and resistive (inclination of the I/V curve) behavior.

The maximum current density, *Jmax*, determined at the plateau starting from −0.1V/SCE, was 2.0 A/m^2^ for the microbial anode established from the control graphite electrode. Jmax was exactly 2.6 A/m^2^ for the treated electrode A and greater than 3.0 A/m^2^ for the treated electrode E. All the Jmax determined from the turn-over CV graphs performed here with a low electrode potential scan rate (1 mV/s) resulted in Jmax values very similar to the Jmax already established from the steady-state CA measurements. Unfortunately, Jmax was not determinable for the A + E electrode, due to its resistive characteristic and high capacitance, both of which masked the interpretation of the faradaic current.

### Comparison of bacterial communities

**Figure 4:**
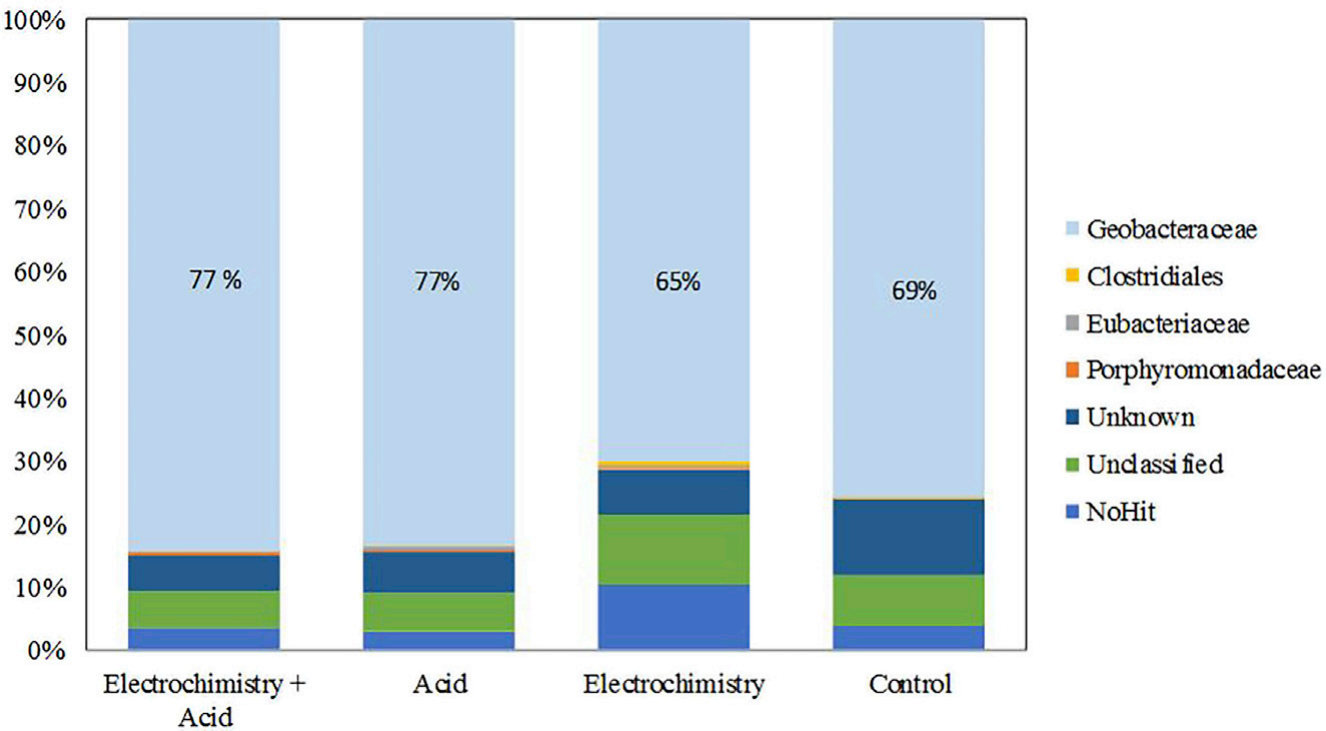
Relative abundance of major bacterial classes identified in the biofilms that had developed after 25 days on A + E, A, E, and control graphite electrodes.

Metagenomic sequencing analysis of bacterial populations was performed on the bacterial communities extracted from the liquid medium (dWW) and from the inoculum (AS) as well as on the electroactive biofilms grown on the four types of electrodes, i.e., treated graphite surfaces (A + E, A, E) and non-treated commercial graphite surface (Control). The details of raw bioinformatics data from the metagenomics pyrosequencing are available in Figure 4. Bacteria from the Geobacteraceae family were present at 77% in the biofilms colonizing the A + E and A electrodes, at 65% for E bioanodes, and at 69% for control bioanodes. In short, the Geobacteraceae family was significantly enriched in biofilm communities of the four bioanodes regardless of the surface condition of the graphite polarized at −0.1 V/SCE. This bacterial family, typically occurring in anodic electroactive biofilms, was only present at 0.06% in the inoculum (AS) and not detected in the liquid medium (dWW). Other bacterial families, much less highly represented, such as Clostridiales, Eubacteriaceae, and Porphyromonadaceae, were also present at less than 1% in all biofilms analyzed, and the remaining families could not be identified.

The predominant bacterial population of the Geobacteraceae family is actually very classical in the field of electroactive biofilms. Examples of diversity analysis on carbon-based anodic bioelectrodes (i.e., carbon cloth, graphite, modified graphite fiber) frequently report a majority of the Geobacteraceae family in biofilms from wastewater (Kiely et al., 2011; Roubaud et al., 2019), marine estuarine sediments (Holmes et al., 2004), the rhizosphere (Kouzuma et al., 2013), or enriched from primary bioanodes (Blanchet et al., 2015). All these cases have been shown independently with natural (Holmes et al., 2004), industrial (Blanchet et al., 2015) and model substrates such as acetate (Kiely et al., 2011) and glucose (Kouzuma et al., 2013). The work of Blanchet et al. (2015) went even further, demonstrating a strong correlation between the current density provided by different bioanodes and the relative abundance of Geobacteraceae.

To sum up, none of the surface treatments had a substantial impact on the population distribution within the bacterial communities that had been established on the electrodes. This was not surprising considering that there were no significant changes in electrode conductivity or hydrophilicity following the treatments (Roubaud et al., 2019). The average steady state current densities obtained with the four types of microbial bioanodes could not be directly correlated with the percentage of Geobacteraceae in the electroactive biofilms.

## Discussion

### Overview of the impact of graphite electrode treatments

The electrochemical (E), acid bath (A), and acid bath + electrochemical (A + E) treatments improved the average steady state current densities produced by microbial bioanodes. The surface area of the post-treatment electrodes was also considerably increased, but the average roughness was maintained at almost the same value (close to 2 μm), so it was certainly nanoporosity and nanotopography that were involved in the increase. The generation of graphene and/or graphene oxides by electrochemical fracturing of the graphite sheets is strongly suspected. This hypothesis is well supported by the increase in the double layer capacity of the treated electrodes. However, this scale of electrode surface modification does not change the developed surfaces accessible to electroactive biofilms. Other, subjacent, explanations would therefore be necessary to justify the doubling of Jmax with the microbial bioanodes that were formed.

A significant change in the surface composition as a result of the treatments was also identified, opening up the possibility of a kind of surface activation, similar to the activation of activated carbon, which increases the adsorption properties of carbonaceous materials. Scenarios favorable to a stronger adsorption of proteins or even cells on the surface of the electrodes, which make the basal layers of the biofilm denser in systems performing EET, can thus reasonably be considered. In the same vein, the organic substrates could be adsorbed and thus concentrated on the electrode surface, but the experiments were carried out with COD concentrations high enough to be definitely non-limiting.

The bacterial community did not differ at all among the biofilms developed on treated or untreated graphite, thus ruling out any hypothesis leading to the selection of different bacterial populations.

### Surface chemistry modification

A + E and E treatments both caused an 18% increase in S_a_ and a 70% decrease in S_ku_ and multiplied the EASA by 54 and 43, respectively. Despite similar topography and specific surface changes, the current density increase was 56% with the E-treated electrode and only 17% with the A + E-treated electrode. The unexpectedly poor performance obtained with the A + E electrode can be explained by the important surface chemistry modifications brought by the A + E treatment. In particular, the addition of O=C-O functional groups that are negatively charged at pH 7 could be detrimental for the adhesion of negatively charged bacteria to the surface because of electrostatic repulsion during early-stage biofilm formation (Byon et al., 2013; Terada et al., 2012). The highly resistive behavior of the A + E-treated electrode observed on CVs both before and after colonization could also be a consequence of this surface chemistry modification. The oxygen-rich group layer could create interferences in the electron transfer between bacteria and the electrode as a result of its low electronic conductivity (Liu et al., 2014; Xie et al., 2010). In the same line of thought, it has been suggested in the literature that a low oxygen content on the anode material may facilitate bacterial attachment, thus favoring the current production of microbial anodes (Cai et al., 2013; Wang et al., 2009).

On the other hand, a higher percentage of nitrogen groups on the surface is generally linked to higher current densities, especially for positively charged N groups, such as amides and ammonium groups, that increase the positive surface charge at pH 7 (Cheng and Logan, 2007; Liu et al., 2014). The increase in positive surface charge favors bacterial adhesion because bacteria are negatively charged, so the surface and bacteria will attract each other electrostatically (van Loosdrecht et al., 1989). In our case, with the A + E treatment, the slight increase of nitrogen percentage did not compensate for the detrimental effect of an increase in the surface oxygen percentage, leading to resistive behavior of the electrode.

### Oxidation of graphite and generation of graphene and graphene oxide

It is well documented that chemical oxidation (i.e., with strong oxidants) or electrochemical oxidation of graphite leads to the generation of graphene and graphene oxide, deforming the arranged structure of the graphite by creating disorder between layers, splitting the spaces between the sheets, and ultimately giving the destructured, porous appearance of oxidized graphite (Ortega Amaya et al., 2017). Methods of electrochemical oxidation of graphite are commonly used with either dilute mineral acid electrolytes or aqueous salt electrolytes (Lowe et al., 2019). They have considerable advantages over purely chemical methods of treatment by bath because they generally take place in a single step, can be carried out in a few minutes or a few hours (unlike chemical methods like Hummers' method and modified Hummers' method [Hummers and Offeman, 1958], which tend to extend over several days), and can be carried out at ambient temperatures, which definitely makes them interesting from an industrial point of view.

Graphene is quite hydrophobic, but it is interesting to note that the oxygen-rich groups attached to the graphene structure make graphene oxide much more hydrophilic. Graphene coating is also used on metallic or conductive materials for hygienizing surfaces and for its anti-biofilm character. Graphene oxide is recognized as an antibacterial agent in its nanoparticulate form. Many studies on hydrogels or composite polymers filled with nanoparticles of graphene and graphene oxide show cytotoxic properties and are markedly detrimental to the survival of bacteria (Tadyszak et al., 2018).

On the surfaces of solid materials, the orientation of the graphene sheets can have a clear influence on the anti-biofilm activity and bactericidal action. Particle orientation parallel to the surface may inhibit bacterial attachment in some cases, but the effect on cell viability is not obvious. For information, single-layer graphene and single-layer graphene oxide have a thickness of about 0.3 nm, which is atomic thickness. For these reasons, when oriented perpendicular to the surface, they are suspected of being capable of breaking the membrane of Gram-positive and Gram-negative bacteria (Notley et al., 2013).

### Modification of nanotopography

Increased surface sharpness (lowered S_ku_) means that there are more topographic elements, such as cavities or peaks, to protect bacteria from shear forces than on a smooth surface, which is beneficial for microbial colonization (Champigneux et al., 2018b; Characklis, 2009).

A + E and E treatments both led to a 16% S_a_ increase, which can be associated with significant increase in the specific surface area, as demonstrated by the BET method measurements and EASA calculations (EASA multiplied by 54 and 43 after A + E and E treatments, respectively). For the A-modified electrode, S_a_ decreased, but its specific surface area increased slightly. In numerous works reported in the literature (Ebrahimi et al., 2017; Liu et al., 2017; Sadri et al., 2017), higher specific surface area basically leads to enhanced performance and the argument that an increase in specific surface area leads to an increase in the amount of electroactive biofilm developed on the surface is widely advanced. However, in most cases, estimating the relevance of linking the increase of specific surface area of the electrodes and the increase in the quantity of electroactive biofilms or bio-electrocatalytic interface according to the scale of surface modification is questionable.

First, it is well known that nanotopography promotes cell adhesion. Second, it is also known to promote the growth of electroactive bacteria by accelerating electron transfer. The recent literature review by Champigneux et al. (2018a, 2018b) states that electrode nanostructuring is more efficient when it is smaller than 300 nm and when a large distance is present between peaks (Champigneux et al., 2018a). However, at this nanoscale, it seems difficult to distinguish the effect of nanotopography of the electrode surface from that of the other interfacial parameters, which are inevitably modified when the nanotopography is modified. The question is still open as to whether the performance of the microbial bioanodes formed from the modified graphites is a direct consequence of the change in the nanotopography of the electrodes or whether the modification of the chemistry or the crystallographic state of the surface has an even more direct effect on the reaction properties, the double layer capacity, or the adsorption properties of the surfaces.

### Electronic property of treated electrodes

A + E, E, and A treated electrodes had capacitive behaviors that the control electrode did not possess. Capacitive bioanodes are an integration of bioanodes with electrochemical capacitors and are capable of storing an electronic charge electrostatically through the formation of a Helmholtz layer by ion adsorption at the electrode/electrolyte interfaces. Two distinct types of capacitive bioanodes are currently described: (1) the bioanode using capacitive or pseudo-capacitive materials as the electrode supporting the anodic electroactive biofilm so that it functions as a true biocapacitor (Caizán-Juanarena et al., 2020; Deeke et al., 2013; Wang et al., 2020), and (2) conventional non-capacitive bioanodes coupled with additional capacitive electrodes giving the assembly a large surface area available for the formation of a double Helmholtz layer (Houghton et al., 2016; Santoro et al., 2016).

The application of these capacitive bioanodes in bioelectrochemical systems has boosted electron recovery processes, with very significant improvements of more than 50% compared with non-capacitive bioanode systems. However, to be effective, a capacitive bioanode must operate intermittently, which means that it must run through a regular charging and discharging process. As Caizán-Juanarena et al. (2020) describe very well, the Helmholtz layer is formed during charging, by a faradaic process of oxidation of organic matter (If) catalyzed by the anodic biofilm, and is released during discharge (Caizán-Juanarena et al., 2020). This electrostatic process produces a non-faradic current called capacitive current (Ic) during discharge.

However, when the potential of the bioanode is kept fixed at −0.1 V/SCE, as in our bioanode formation tests, the Helmholtz layer theoretically reaches a steady state fairly quickly, before any exchange current is measured between the working and auxiliary electrodes. The state of the Helmholtz layer no longer changes if the interface does not change, e.g., by the formation of a biofilm, or if the chemical composition of the electrolyte or the potential of the electrode does not change. Thus, at a constant imposed potential, the purely capacitive nature of the electrode could not explain the improvement in electrochemical behavior observed with bioanodes formed from treated graphite electrodes.

The current density increase that was obtained with the A + E-, E-, and A-treated electrodes is not directly caused by the increased capacitance but by the increased specific surface area. So, in our case, the increased capacitance is certainly a consequence of the increased specific surface.

### Validation of the similarity of the experimental interface conditions

In contrast to conventional biofilms that form on non-conductive solid surfaces, electroactive biofilms have the special feature of additionally using the electrode surface as an exogenous respiration pathway. In view of this dual functionality of the electrode surface, it has been determined that EA biofilms are, nevertheless, viable in their entirety and metabolically active over their entire thickness (Franks et al., 2010; Semenec and Franks, 2015). Microorganisms distant from the electrode then use the electrode remotely via interconnected cell-to-cell or cell-to-electrode networks of conductive pili or endogenous redox shuttles that diffuse into the biofilm (Santoro et al., 2017). Cells in direct contact with the electrode or in direct contact with other microbial cells manage electronic exchanges through EET mechanisms involving cytochrome-like membrane proteins (Patil et al., 2012).

Cell interactions are therefore multiple within electroactive biofilms, and they can be chemical (substrates, nutrients, redox products such as protons, quorum-sensing molecules, etc.), physical (electrode, hydrodynamics, temperature, light), or electronic (EET mechanisms). There are then several selective pressure drivers leading to a more or less successful enrichment in electroactive bacterial species from a mixed population of microorganisms. They can be categorized according to whether they are related to the electrode or to the electrolyte. Concerning the electrode, the notion of biocompatibility is primordial. Then comes the electrode material, i.e., its chemical composition, and its surface properties in terms of topography, wettability, electrical conductivity, and reactivity. The potential of the electrode is also a determining factor in the predominance of electroactive populations within the electroactive biofilm because this potential of the solid electron acceptor theoretically conditions the Gibbs free energy gained for the electroactive microorganisms (Dennis et al., 2016; Wagner et al., 2010). Concerning the electrolyte, conductivity, pH, or the presence of soluble electron acceptors (especially O_2_, NO_3_^-^, NO_2_^-^, Fe^3+^) can impact the selection of specifically adapted populations within a microbial community. More importantly, the concentration and type of the electron-donating anodic substrate also plays a major role in determining the structure of the microbial community and the electrical production of anodic biofilms of mixed species (Chae et al., 2009; Ullah and Zeshan, 2019).

To compare electrode materials, it therefore seems essential to ensure that the vast majority of effectors that may affect the establishment of different communities are precisely controlled experimentally and identical from one experiment to another (Pocaznoi et al., 2012b). In the work presented here, all the experiments were conducted in parallel, i.e., at the same temperature, in identical configurations of electrochemical bioreactors, and using the same electrolytic reaction medium composed of real deoxygenated wastewater effluent. For the comparison of the electrodes, all the electrode samples had the same geometry. Their position relative to the auxiliary and reference electrodes was reproducible in all tests, and, of course, the electrode potential was consistently applied at −0.1 V/SCE. Taking all these precautions together justifies the interpretation that the differences in electrochemical behavior are really due to different interactions at the interface between the electrode and the electrolyte. The other less elegant possibility would be to carry out a number of tests that were less controlled but more numerous and then to perform a deep statistical analysis of these replicates (Larrosa et al., 2009).

Not surprisingly, the bacterial communities identified on the different flat graphites, modified or unmodified, were ultimately very similar. Insofar as the surface treatments did not impact either the conductivity or the wettability of the graphite, the few instances of predominance that differ in the populations can only be justified, if significant, by a change in the surface composition of the graphite.

### Ranking analysis of the general performance of graphite-modified bioanodes in the bioelectrochemical systems fed with real wastewater

In this study, a maximum stabilized current density of 2.8 A/m^2^ was obtained through the electrochemical surface treatment of a graphite electrode. This study was conducted exclusively with real dWW. Wastewater currently used in bioelectrochemical systems (mainly microbial electrolysis cell and microbial fuel cell) can be divided into two categories: dWW and industrial wastewater from a wide variety of sources (breweries, dairies, refineries, etc.). The two highest current densities obtained with these real effluents are 10.7 A/m^2^ and 10.3 A/m^2^. These performances were obtained, respectively, with biorefinery wastewater (Pannell et al., 2016) and brewery wastewater (Yu et al., 2015). With dWW, the highest current densities obtained are 3.8 A/m^2^ (Ullery and Logan, 2015) and 3.5 A/m^2^ (Blanchet et al., 2016), but these current densities were achieved with electrodes having three-dimensional geometry: a graphite fiber brush and carbon cloth, respectively. The average current densities calculated from the values presented in 48 publications are 2.6 A/m^2^ for industrial wastewater and 0.8 A/m^2^ for dWW. This significant difference between the average current densities is partly due to the fact that industrial wastewater is generally more conductive than dWW. The conductivity of dWW is 1.5 mS/cm on average, whereas that of industrial wastewater can reach 7 mS/cm (Yen et al., 2016). Industrial wastewater generates less resistance to solution charge transfer in MFC and MEC reactors, thus minimizing ohmic drop losses. Moreover, the COD (i.e., organic matter concentration) of industrial wastewater is between 5,000 and 12,000 mg/L (Rajeshwari et al., 2000), whereas that of dWW is between 320 and 740 mg/L (Almeida et al., 1999), about 16 times less. This significant difference has an impact on bioanode kinetics, which, we recall, is generally dependent on the organic matter concentration in the medium (Peng et al., 2013; Roubaud et al., 2019).

The materials most commonly used for bioanodes are carbon-based, and are chosen for their high biocompatibility. Carbon anodes can take several forms. Carbon fiber brushes are frequently used both at the laboratory scale (Kiely et al., 2011; Lu et al., 2016; Ullery and Logan, 2015) and at the industrial scale (Cusick et al., 2011). This anode geometry is interesting because the fibers allow a high surface/volume ratio, which makes it possible for a large number of bacteria to adhere to and interact with the anode. Carbon felt is also widely used as it has the advantage of being easily adaptable to all reactor geometries (Escapa et al., 2012; Gil-Carrera et al., 2013; Moreno et al., 2016; Rabaey et al., 2010). It is very porous and thus has a large specific surface area. Nevertheless, it has been shown that, when a complex inoculum (activated sludge) and a real substrate such as wastewater or biowaste is used, the felt colonization thickness is very small compared with the total thickness of the felt (400 μm for 0.8 cm thickness) (Blanchet et al., 2016). Graphite in the form of plates (Samsudeen et al., 2016), granules (Ditzig et al., 2007), or rods (Liu et al., 2004) can also be used as a support for anodic electroactive biofilm. Graphite has the advantages of being inexpensive and having high conductivity compared with carbon fiber materials (brushes, cloth, felt). It is a material with a low specific surface area, but this disadvantage can be compensated by the creation of three-dimensional anode geometries through machining and assembly of commercial graphite plates or rods. In a few cases, metallic materials have been used: a tin-coated copper grid (Çetinkaya et al., 2015) and a stainless-steel grid (Behera et al., 2010). The current densities achieved were 0.6 and 0.16 A/m^2^, respectively, below the average of 0.8 A/m^2^. In contrast, highly conductive, metallic materials are not very biocompatible, and some, such as copper, have poor chemical stability in aqueous media at neutral pH. In addition, copper ions are harmful to microorganisms.

### Conclusion

The electrochemical treatments of a graphite electrode studied here multiplied the EASA by 43. Treatment in a mild pH phosphate buffer did not significantly change the surface chemistry of the graphite. In a bioelectroanalytical system allowing optimal control of the interfacial conditions, this electrochemically treated graphite led to the development of bioanodes fed only with dWW that were capable of continuously supplying 2.8 A/m^2^. This current density is 56% higher than that of a microbial anode established on an untreated graphite electrode under the same conditions, and more than three times the average current density of 0.8 A/m^2^ reported by a panel of 48 articles from the literature focused on bioanodes fed with dWW. This increase in current was attributed to changes in graphite surface nanotopography improving bacterial attachment and increasing the specific surface area of the graphite electrode and EASA. The communities of bacteria that colonized the modified and unmodified electrodes were found to be very similar as the interfacial conditions were assumed to be very similar due to the electro-analytical experimental system developed specifically for this study.

Electrochemical treatment is a simple but effective way to improve the performance of graphite bioanodes and could be easily adapted to the treatment of electrodes on a large scale thanks to the fact that industrial electrolytic surface treatment plants are already widespread. On the other hand, performing the same treatment in a highly concentrated acid solution has also led to changes in the surface chemistry that are detrimental to bacterial adhesion and which have limited the current density produced by the bioanodes having undergone this treatment. The bacterial community of the biofilm was not impacted, only the amount of biofilm adhering is questioned at this stage.

### Limitations of the study

The surface treatment of graphite electrode that allowed the highest current density improvement created beneficial physical surface modifications and no detrimental chemical surface modifications. The increase in current densities produced by surface-treated bioanodes is particularly due to the increased ESEA of the electrodes following electrochemical treatments.

All these observations have been experimentally validated with a single system of anodic biofilms formed from the electroactive endogenous flora of real dWW. The behavior of these electrodes with an optimized surface would deserve to be validated using alternative inocula of electroactive bacterial populations, either with model strains of the *Geobacter* or *Shewanella* genera, or complex communities such as sediments, soils, or digester sludge.

Also, the electrochemical modification of graphite is based here on an empirical method that consists of 24 successive cycles of constant potential fixed at +1.5 V/SCE for 1 h and a potential scan from −1.0 V/SCE to +1.0 V/SCE at 30 mV/s (Cercado-Quezada et al., 2011). A better understanding of the independent effect of the potential boundaries, the scan rate, or the fixed potential part would allow further optimization of the processing. The conversion to an electrochemical galvanostatic treatment method would also be a further step toward simplifying and industrializing the surface treatment process.

### Resource availability

#### Lead contact

Further information and requests for resources should be directed to and will be fulfilled by the Lead Contact, Benjamin Erable (benjamin.erable@ensiacet.fr).

#### Materials availability

Our work did not generate any new unique reagents.

#### Data and code availability

Our work did not report any unpublished custom code, software, or algorithm.

## Methods

All methods can be found in the accompanying Transparent Methods supplemental file.

## Supplemental information

Document S1. Transparent methods and Figure S1
