# Supplementary material for: Industrially scalable surface treatments to enhance the current density output from graphite bioanodes fueled by real domestic wastewater
Source: iScience. 2021 Feb 7;24(3):102162. doi: 10.1016/j.isci.2021.102162 (PMC7907815; doi:10.1016/j.isci.2021.102162)
Supplement: Document S1. Transparent methods and Figure S1 [file mmc1.pdf]

iScience, Volume *24*

## Supplemental information

# Industrially scalable surface treatments

# to enhance the current density output from graphite

# bioanodes fueled by real domestic wastewater

**Emma Roubaud, Rémy Lacroix, Serge Da Silva, Jérôme Esvan, Luc Etcheverry, Alain Bergel, Régine Basséguy, and Benjamin Erable**

# Supplemental figures

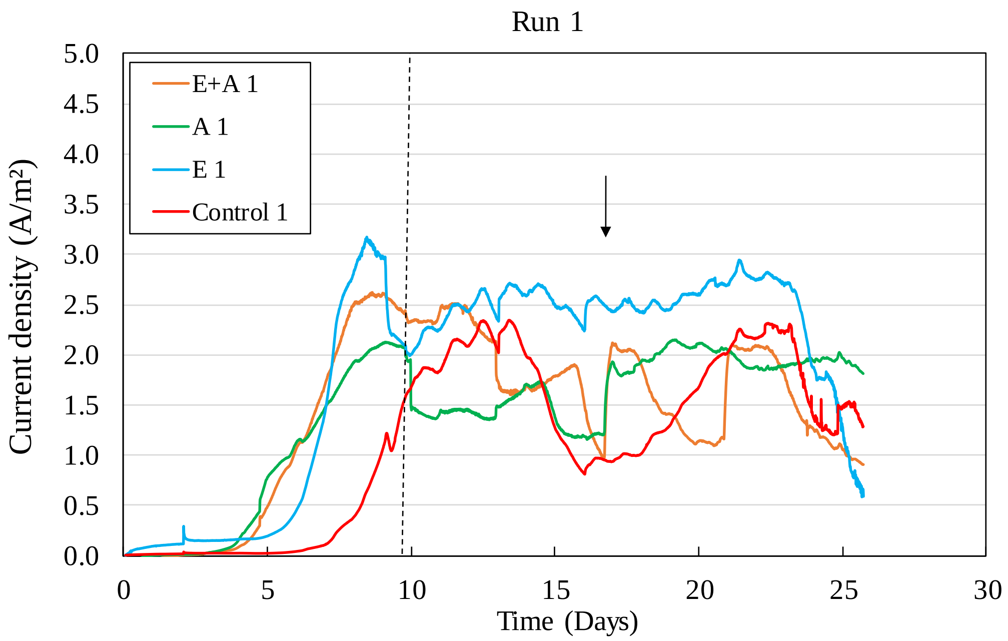


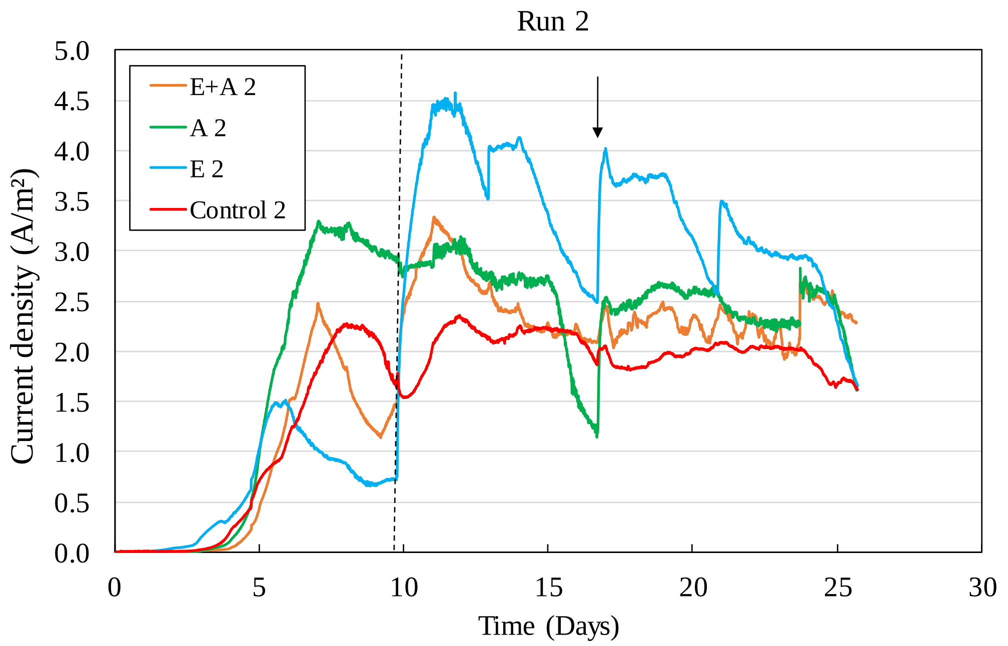


Figure S1. Chronoamperometries for dWW-fed graphite bioanodes (duplicates), related to Table 5. The reactors were initially run in batch mode for 10 days and were then switched to recirculation mode (dashed line) in closed loop with a 2 L tank of hydrolyzed dWW that was renewed at day 17 (black arrow).

# Transparent methods

## 1 Graphite anode treatments

The acid bath treatment (**named A**) was performed by soaking the graphite plates in an aqueous solution containing 4 M nitric acid and 1.5 M sulfuric acid for 24 h (Li et al., 2014). The electrochemical treatment was performed directly using the 3-electrode set-up described previously. This treatment consisted of 24 successive cycles of constant potential fixed at +1.5 V/SCE for 1 h and a potential scan from −1.0 V/SCE to +1.0 V/SCE at 30 mV/s (Cercado-Quezada et al., 2011). This treatment was performed either in 50 mM phosphate buffer at pH 6.7 (electrochemical treatment only, **named E**) or in an acid bath containing 4 M nitric acid and 1.5 M sulfuric acid (combined acid and electrochemical treatment, **named A+E**).

## 2 Material surface analysis

### 2.1 SEM observations

Treated and untreated surfaces were observed with a LEO 435 VP high-performance, variable pressure scanning electron microscope (SEM) having a maximal resolution of 4.0 nm.

### 2.2 Surface roughness measurements

Surface roughness was measured with an optical microscope (S-Neox, Sensofar) with the focus variation method. Two surface roughness criteria were considered to evaluate the surface topology: (1) the mean arithmetic height, $S_a$, and (2) the surface flattening factor, Kurtosis, $S_{ku}$, which gives an idea of the abruptness of the surface. A Gaussian distribution of peaks on the surface is characterized by a kurtosis value of 3. Typically, spiky surfaces have $S_{ku}$ values over 3 and non-abrupt surfaces have $S_{ku}$ values under 3 (Voegel et al., 2020).

### 2.3 Photoelectron emission spectra analysis

The photoelectron emission spectra were recorded using a monochromatized Al K-alpha (hv = 1486.6 eV) source on a Thermo Scientific K-Alpha system. The X-ray spot size was about 400 µm. The pass energy was fixed at 30 eV with a step of 0.1 eV for core levels and 160 eV for surveys (step 1 eV). The spectrometer energy was calibrated using the Au $4f_{7/2}$ (83.9 ± 0.1 eV) and Cu $2p_{3/2}$ (932.8 ± 0.1 eV) photoelectron lines. XPS spectra were recorded in direct mode N (Ec) and the background signal was removed by using the Shirley method.

The flood gun was used to neutralize charge effects on the top surface.

## 2.4 Specific surface evaluation of graphite electrodes

Specific surface measurements were performed with the BET method on a Belsorp-Max system (Bel, Japan) with nitrogen gas.

The electrochemically accessible surface area (EASA) of each type of electrode was estimated by means of cyclic voltammetry (CV). This electrochemical technique allows the phenomena taking place at the material-solution interface to be determined. In particular, it gives access not only to the faradic reactions but also to the capacitive and/or resistive character of the system under study. As the currents are proportional to the reactive surface area, it is possible to evaluate this area by comparing the current found with the treatment electrodes to that of the graphite control electrode.

# 3 Bio-electrochemical setup

Bio-electrochemical experiments were performed in 3-electrode setups. The working electrodes were 2 cm x 1 cm graphite plates so the surface area used to calculate current densities was 4 $cm^2$ (taking both sides of the sample into account). The electrodes were connected to titanium rods used as current collectors. The counter-electrodes (cathodes) were stainless steel grids arranged in a circle around the working electrodes and a stainless steel wire served as the current collector. The reference electrodes were saturated calomel electrodes (SCE). The 3-electrode setups described were placed in 600 mL glass reactors.

The 600 mL bio-electrochemical reactors were initially filled with a dWW and inoculated with 5% v/v of activated sludge (AS). A continuous flow of nitrogen gas (5 mL/s) was added to maintain anoxic conditions. dWW and AS were collected at a local sewage treatment plant (Castanet-Tolosan, France) and stored at 4°C before use in the reactors. The organic matter present in the wastewater was the only anodic substrate supplied to the EA biofilms. No synthetic substrates were used in the experiments. The concentration of organic matter in the wastewater is expressed in relation to the Chemical Oxygen Demand (COD) here. The total COD ($COD_T$) was the sum of two fractions: the soluble COD ($COD_S$), corresponding to small organic molecules easily degradable by bacteria (acetate, formate, propionate, etc.) and the particulate COD ($COD_P$), corresponding to organic matter that was inert or difficult to biodegrade. The $COD_S$ was always higher than 400 mg/L to ensure that conditions were non-limiting in anodic substrate (i.e. fuel), and the maximum COD has never exceeded 650 mg/L. The conductivity of the domestic wastewater was around 1.2 mS/cm and the initial pH was between 7.2 and 7.6.

Anodes were polarized at -0.1 V/SCE and the current was recorded every 10 min. Cyclic voltammetry (CV) was recorded at 1 mV/s in the −0.5 to +0.2 V/SCE range at the beginning and end of the

experiments. The reactors were operated for 10 days in batch mode and were then switched to recirculation mode (1.5 mL/min) with 2 L tanks of dWW hydrolyzed for 7 days in anoxic conditions. The tanks were renewed after 7 days. This protocol was designed to ensure a high, constant COD inflow to the bioanodes so that they would produce stable current densities. It is described more thoroughly in Roubaud et al. (2019) (Roubaud et al., 2019). All the experiments were conducted in duplicate.

## 4 Bacterial community analysis

The graphite bioanodes were placed in plastic tubes with 50 mL of phosphate buffer and put into an ultrasonic bath at 80 W for 30 min to detach the biofilm. Two 50 mL dWW and AS samples were also prepared. The tubes were centrifuged for 15 minutes at 4600 g at 6 °C. After the supernatant had been discarded, a DNA extraction kit (DNeasy PowerBiofilm, Qiagen) was used on the pellets, following the manufacturer's recommendations. The DNA concentrations were checked with absorbance at 260 nm and any possible contamination by proteic and humic acid was detected by absorbance at 280 nm and 230 nm respectively. The DNA samples were sent to RTLab (Texas, USA), where the DNA was amplified by PCR and sequenced with the bacterial primers 28F (5'- GAG TTT GAT YMT GGC TC -3') and 519R (5'- GWA TTA CCG CGG CKG CTG -3') according to RTLab protocols. Data analysis for DNA quality, DNA sequence alignment, and clustering in operational taxonomic units and taxonomic assignment were also performed by RTL according to their protocol (available at http://rtlgenomics.com/). The full, more detailed procedure is available in Blanchet et al. (2015)(Blanchet et al., 2015).

# Supplemental references


Blanchet, E., Desmond-Le Quemener, E., Erable, B., Bridier, A., Bouchez, T., Bergel, A., 2015. Comparison of synthetic medium and wastewater used as dilution medium to design scalable microbial anodes: Application to food waste treatment. Bioresour. Technol. 185, 106–115.

Cercado-Quezada, B., Delia, M.-L., Bergel, A., 2011. Electrochemical micro-structuring of graphite felt electrodes for accelerated formation of electroactive biofilms on microbial anodes. Electrochem. commun. 13, 440–443.

Li, Baitao, Zhou, J., Zhou, X., Wang, X., Li, Baikun, Santoro, C., Grattieri, M., Babanova, S., Artyushkova, K., Atanassov, P., Schuler, A.J., 2014. Surface modification of microbial fuel cells anodes: Approaches to practical design. Electrochim. Acta 134, 116–126.

Roubaud, E., Lacroix, R., Da Silva, S., Etcheverry, L., Bergel, A., Basséguy, R., Erable, B., 2019. Benchmarking of Industrial Synthetic Graphite Grades, Carbon Felt, and Carbon Cloth as Cost-Efficient Bioanode Materials for Domestic Wastewater Fed Microbial Electrolysis Cells. Front. Energy Res. 7, 1–12.

Voegel, C., Durban, N., Bertron, A., Landon, Y., Erable, B., 2020. Evaluation of microbial proliferation on cementitious materials exposed to biogas systems. Environ. Technol. 41, 2439–2449.
